# Effects of a false-positive result in newborn congenital hypothyroidism screening on parents in Guangxi, China

**DOI:** 10.3389/fped.2023.1134923

**Published:** 2023-05-11

**Authors:** Si-Jing Tu, Yu-Jia Wei, Bu-Tong Chen, Xiao-Fei Zhang, Chao Luo, Bai-Qing Dong

**Affiliations:** ^1^ School of Public Health and Management, Guangxi University of Chinese Medicine, Nanning, China; ^2^ School of Public Health, Hangzhou Normal University, Hangzhou, China; ^3^ School of Humanities and Social Sciences, Guangxi Medical University, Nanning, China; ^4^ Department of Pediatrics, The Maternal and Child Health Hospital of Guangxi Zhuang Autonomous Region, Nanning, China; ^5^ Shanghai Mental Health Center, Shanghai Jiao Tong University School of Medicine, Shanghai, China

**Keywords:** newborn screening, false-positive, congenital hypothyroidism, parenting stress index, health education

## Abstract

**Background:**

As more than 500,000 neonates participate in newborn congenital hypothyroidism (CH) screening in Guangxi Zhuang Autonomous Region each year, the overall number of false-positive (FP) cases has increased. We aim to assess the parental stress in parents of neonates with FP CH results in Guangxi, find out the influence factors related to demographics, and provide the basis for personalized health education.

**Methods:**

The parents of neonates with FP CH results were invited to participate in the FP group, and the parents of neonates with all negative results were invited to participate in the control group. The parents completed a questionnaire on demographics, knowledge of CH, and the parental stress index (PSI) in the hospital for the first time. The follow-up visits for PSI were conducted 3, 6, and 12 months afterward through telephone and online.

**Results:**

A total of 258 and 1,040 parents participated in the FP and control groups, respectively. The parents in the FP group had better knowledge of CH and higher PSI scores than the parents in the control group. The result of logistic regression showed that the major influence factors related to the knowledge of CH were FP experience and source of knowledge. The parents in the FP group who were well-informed during the recall phone call had lower PSI scores than the other parents. The parents in the FP group showed decreasing PSI scores gradually in follow-up visits.

**Conclusion:**

The results suggested that FP screening results may affect parental stress and parent–child relationship. FP results increased the stress on the parents and increased their knowledge of CH passively.

## 1. Introduction

Newborn screening (NBS) is a public health program that enables the presymptomatic identification and early treatment of certain diseases and disorders in the first weeks of life (1). To identify such diseases and disorders, NBS debuted in the USA in 1961 and was first introduced in China in the early 1980s (2). The Guangxi Zhuang Autonomous region is located in southern China, with more than 56.95 million people of various ethnicities and cultures. The first newborn screening center was established in Guangxi in 2005, there are more than 10 centers throughout Guangxi now, and they are responsible for screening more than 500,000 blood samples yearly. The biggest NBS center in Guangxi is the Guangxi NBS Center (called GX-NBSC for short), with a sample size of over 200,000 per year.

Although the testing programs and testing protocols differed among provinces, congenital hypothyroidism (CH) and phenylketonuria (PKU) were the most commonly screened programs in China (3). The NBS programs in Guangxi include CH, PKU, congenital adrenal hyperplasia (CAH), glucose-6-phosphate dehydrogenase (G6PD) deficiency, thalassemia, congenital deafness, and inherited metabolic diseases (IMDs). Thalassemia and G6PD deficiency are strongly recommended programs in Guangxi due to their high prevalence; however, abnormal NBS results in most neonates were mild and not life-threatening (4, 5). CH, a congenital metabolic dysfunction, is a thyroid hormone deficiency that becomes apparent after birth. Severe CH can lead to growth failure and permanent intellectual disability, significantly impacting the child and family (6). Previous studies have reported that the prevalence of CH in Guangxi is 1/1,694, which is slightly higher than the average level around the world (7). Early diagnosis and timely intervention will yield better prognoses and reduce healthcare costs for CH patients.

In line with standard practice worldwide, neonates with positive NBS results were recalled for follow-up tests and asked for a pediatrician consultation as soon as possible in China (8). A previous study showed that some parents who receive a positive result of metabolic disease screening experience a long-lasting psychological change, even several months after discovering that the result was a false-positive (FP) (9). However, few studies concern the psychological effect of parents who received FP NBS results for CH in China, let alone in Guangxi. In Guangxi, the cutoff value of thyroid-stimulating hormone (TSH) is 8 uIU/ml, and the rate of FP is approximately 1% in GX-NBSC; thousands of neonates with an elevated TSH level in the initial NBS test have to be recalled each year (200,000 neonates for NBS × 1%) (10). In this study, the long-term psychological effects of FP results in NBS for CH and its cross-sectional relevant knowledge on parents of Guangxi were evaluated by questionnaires to provide targeted health education and reduce psychological stress.

## 2. Materials and methods

### 2.1. Study design, recruitment, and data collection

**Figure 1:**
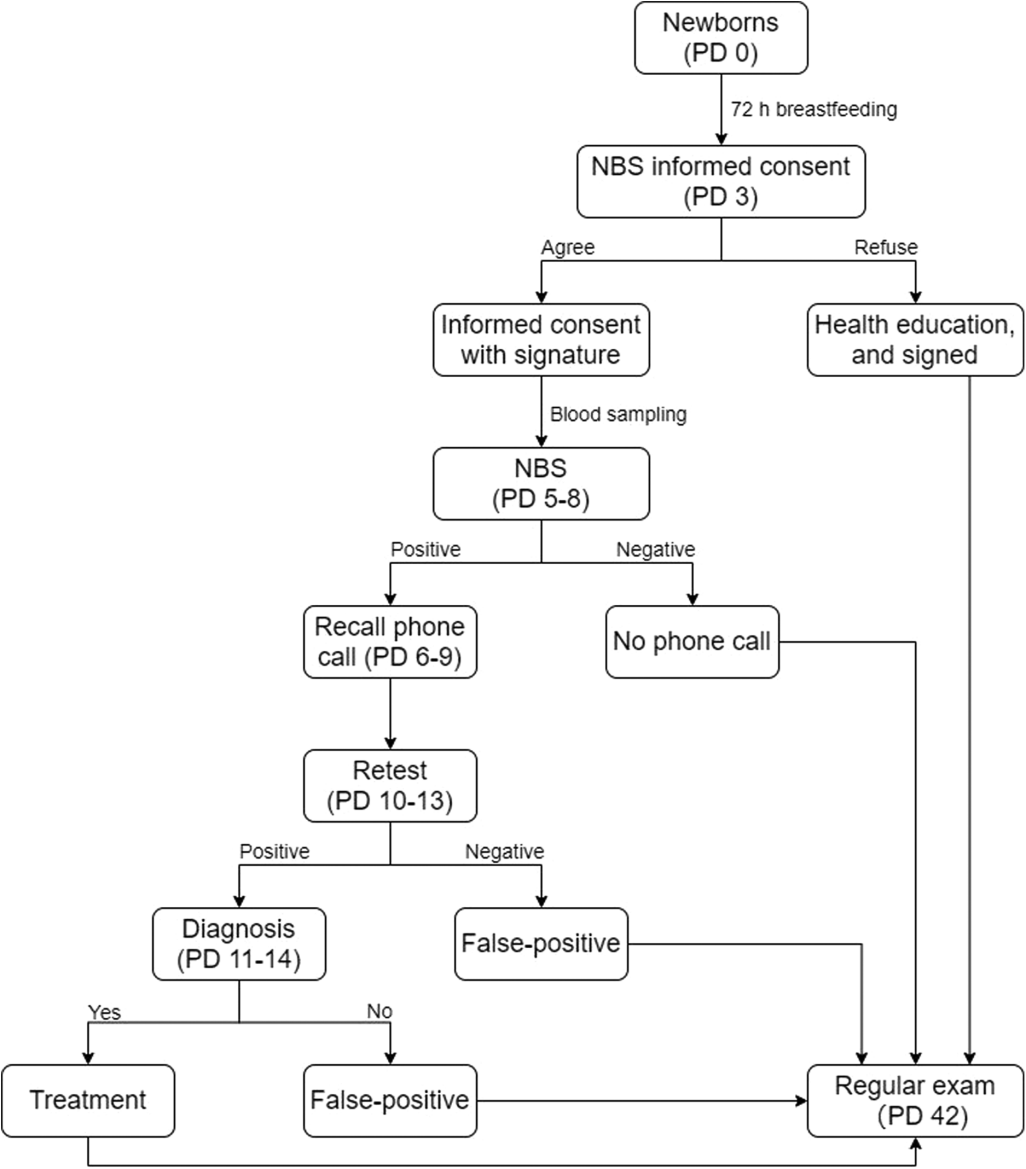
Flowchart of newborn screening in GX-NBSC. PD, postnatal day.

In China, the standard flow of NBS (Figure 1) is as follows: (a) collect a heel-prick blood sample 72 h after first breastfeeding (no later than the first week); (b) perform the NBS and provide the results within 10 days; (c) make a phone call to recall the neonate immediately if abnormality (e.g., positive result, contaminated blood spot) occurs; and (d) the first physical exam for neonates is recommended at 42 days after birth. The parents whose neonates had abnormal NBS results were asked for a retest in GX-NBSC as soon as possible, and the retest result would be provided within 24 h. The parents would not receive phone calls from GX-NBSC if the initial NBS results were within normal reference ranges, and most of them preferred to fetch the report during the first physical exam for neonates. First, we conducted our first investigation in GX-NBSC during the first physical exam (postnatal day 42) by face-to-face interviews and written questionnaires. Due to the COVID-19 pandemic in China, the follow-up visits were conducted through telephone and online survey platform WenJuanXing (WJX, https://www.wjx.cn/) in 3, 6, and 12 months afterward. WJX is a professional online questionnaire survey, examination, evaluation, and voting platform that provides users with robust and humanized online questionnaire design, data collection, custom reports, and survey result analysis.

From 01 September to 30 October 2020, parents of neonates were invited to participate in our investigation by referral of the pediatricians in GX-NBSC; then, the parents who met the inclusion criteria but did not meet the exclusion criteria were interviewed by researchers. For a family, either the mother or father was invited. The inclusion criteria for the FP group were the parents of neonates whose initial result was abnormal or inconclusive for CH screening and negative in a follow-up retest; the inclusion criteria for the control group were the parents of neonates whose initial results were all within the normal reference ranges. The exclusion criteria are as follows: (a) mothers of neonates with thyroid malfunction or mothers who took drugs that can affect thyroid function during pregnancy, (b) parents of neonates who were born at less than 32 weeks of gestation or diagnosed with severe health problems, and (c) parents of neonates were unable to communicate. Furthermore, invalid written questionnaires were excluded from the following analyses, which included overfill or missing answers to some questions. All the participants signed the informed consent forms. This study was approved by the Institutional Review Board of the Guangxi University of Chinese Medicine (No. GXUCM_IRB_H_2019-11-01-1). Participants or the public were not involved in the design, conduct, reporting, or dissemination plans of our research.

### 2.2. Data collection

The first interview was completed and tape-recorded by our well-trained nurses. The questionnaires were scored by three researchers who were blinded to the group identities. The questionnaire consisted of the following three sections.

#### 2.2.1. Demographics

This section was designed to collect demographic data, including sex, age, ethnicity, education year, annual household income, and living area.

#### 2.2.2. Knowledge of CH

In this section, 10 questions included the knowledge on the causes, symptoms, treatment, and diagnosis of CH and one multiple-choice question for the source of knowledge. This knowledge survey was established by our academic board, which includes clinicians, public health experts, genetic counselors, clinical laboratory technicians, and statisticians. As a result, one point is given for a correct answer, and a zero is given for a wrong answer. The parent who got six points or more was considered to have enough relevant knowledge of CH; otherwise, they were considered insufficient (score range: 0–10). The cutoff value (six points) was decided based on our presurvey. Parents were asked to explain the reasons for reducing the probability of answering correctly by chance. After the interview, the pediatricians would provide health education on the wrong questions.

#### 2.2.3. Parenting stress

In this part, parents had to answer the questions in the parenting stress index-short form (PSI-SF) (Chinese version) to assess their stress. The PSI-SF (Chinese version) has been described previously (11), which includes 36 items and provides a total stress score with 3 subscale scores on the following domains: parental distress, parent–child dysfunctional interactions, and difficult child behavior. Each item was rated on a 5-point Likert scale, with responses ranging from totally agree to totally disagree. The normal range for the total stress score is 55–85; scores greater than 85 indicate that the parent needs clinical treatment, while scores less than 10 indicate that the result is questionable. If the parent scored greater than 85 points, a psychiatrist was referred to provide psychological help. In the follow-up visit, the researchers first called the parents in the FP group for further consent and then offered a link to PSI-SF in WJX.

### 2.3. Statistical analysis

The quantitative data are presented as means ± SD; the descriptive data are presented as frequencies and percentages. IBM SPSS Statistics software (version 26.0, Chicago, USA) was used for data analysis. Categorical variables were expressed as percentages, and significance was assessed by the *χ*^2^ test. The age of the parents was analyzed by Student's unpaired *t*-test, and other demographic characteristics were analyzed by using the Wilcoxon rank-sum test for continuous and scale variables and Fisher's exact test for dichotomous variables. Student's unpaired *t*-test was also used to compare the knowledge of NBS and PSI scores between groups. For the PSI scores, subjects who failed the defensive response index (<10) were excluded from the analyses, and analysis of variance (ANOVA) was used to compare the PSI score in the FP group among follow-up visits. All *P* values were two-sided, and values <0.05 were considered significant.

## 3. Results

### 3.1. Sample characteristics

**Figure 2:**
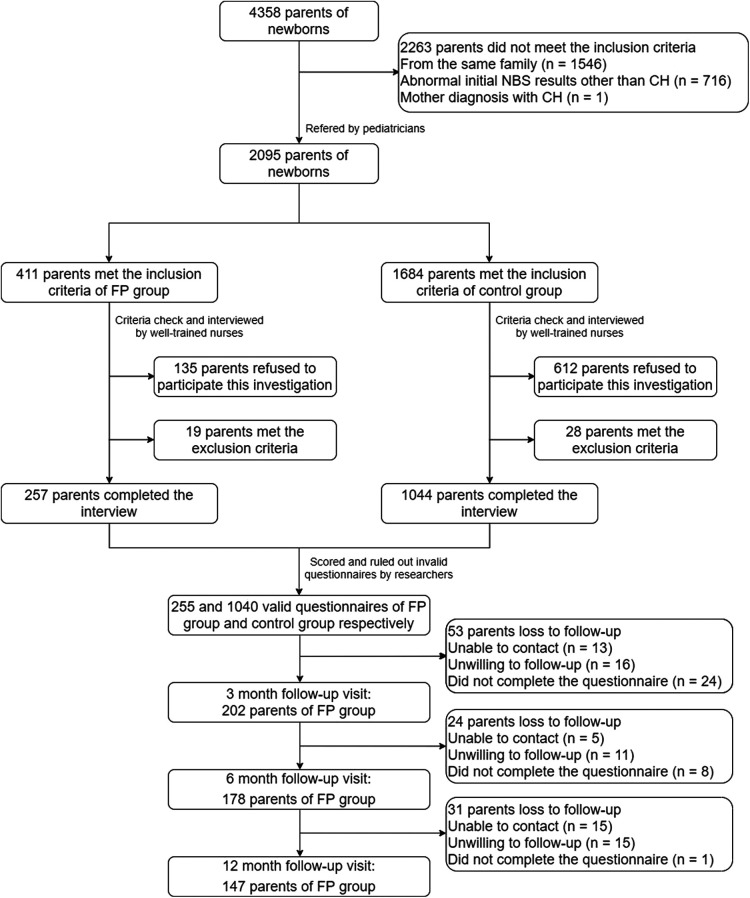
Flowchart of this study.

During our first survey, 411 parents met the inclusion criteria for the FP group, 1,684 parents met the inclusion criteria for the control group, 133 and 612 parents refused our invitation, respectively, and 19 and 28 parents met the exclusion criteria, respectively. After ruling out the invalid questionnaires (e.g., overfill or missing answers to some questions), 258 and 1,040 valid questionnaires of the FP group and control group, respectively, were analyzed in this study. In 3, 6, and 12 months follow-up visits, 202, 178, and 147 parents of the FP group participated, respectively (Figure 2). The characteristics of the participants are reported in Table 1. The FP group included 121 mothers and 137 fathers of 124 daughters and 134 sons. The average age of the parents was 28.87 ± 5.84 years. In the control group, 500 mothers and 540 fathers of 509 daughters and 531 sons were included. The average age of the parents was 28.98 ± 5.32 years old. The sex, age, ethnicity, number of children, education year, living area, and annual household income had no statistical difference between these two groups (all *P*’s > 0.05). Thus, the family demographic variables did not need to be controlled for in subsequent analyses.

**Table 1:** Demographic characteristics of participants [means ± SD or *N* (%)].

Variable	FP group (*n* = 258)	Control group (*n* = 1,040)	*χ^2^*/*t*	*P*
Age of parents (years)	28.87 ± 5.84	28.98 ± 5.32	0.29	0.770
Parent female sex (%)	121 (46.9)	500 (48.1)	0.11	0.735
Neonate female sex (%)	124 (48.1)	509 (48.9)	0.06	0.800
First child (%)	201 (77.9)	792 (76.2)	0.353	0.552
Ethnicity				
Han	128 (49.6)	524 (50.4)	1.73	0.479
Zhuang	123 (47.7)	472 (45.4)		
Other	7 (2.7)	44 (4.2)		
Years of education				
≤9	83 (32.2)	391 (37.6)	5.04	0.080
10–14	100 (38.7)	329 (31.6)		
>14	75 (29.1)	320 (30.8)		
Living area				
Rural	145 (56.2)	517 (49.7)	3.48	0.062
City	113 (43.8)	523 (50.3)		
Annual household income (yuan)				
<50,000	74 (28.7)	316 (30.4)	0.47	0.792
50,000–100,000	145 (56.2)	560 (53.8)		
>100,000	39 (15.1)	164 (15.8)		

FP, false-positive.

### 3.2. Relevant knowledge of CH

The parents in the FP group had a higher correctness rate for every question than those in the control group, especially in the clinical symptoms and treatment and prevention parts (all *P*’s < 0.001). The parents in the FP group had higher scores than the parents in the control group (*t* = 20.26, *P* < 0.001), which indicated that they had more relevant knowledge of CH. According to our criteria (six or more points), 324 parents were considered to have enough relevant knowledge of CH, which was 140 in the FP group (54.26%) and 184 in the control group (17.69%) (Table 2). Within the FP group, the demographic factors were not associated with having enough relevant knowledge of CH (all *P*’s > 0.05).

**Table 2:** Parents’ awareness of relevant knowledge of congenital hypothyroidism (CH) [means ± SD or *N* (%)].

Questions (correct answer)	FP group (*n* = 258)	Control group (*n* = 1,040)	*χ^2^*/*t*	*P*
Causes				
The incidence of CH may be associated with the father's smoking and drinking behaviors—answered No (%)	106 (41.1)	364 (35.0)	3.31	0.069
The incidence of CH may be associated with the maternal iodine intake—answered Yes (%)	145 (56.2)	519 (49.9)	3.28	0.070
The incidence of CH is totally determined by genes—answered No (%)	134 (51.9)	457 (43.9)	5.33	0.021
Clinical symptoms				
Most newborns have no obvious clinical symptoms of CH initially—answered Yes (%)	224 (86.8)	156 (15.0)	515.0	<0.001
No clinical symptoms means no harm to CH children—answered No (%)	173 (67.0)	105 (10.1)	398.5	<0.001
CH is an endocrine disease and does not affect children's intelligence—answered No (%)	202 (78.3)	87 (8.4)	584.1	<0.001
Treatments and Preventions				
Thyroidectomy can cure CH for good—answered No (%)	93 (36.0)	211 (20.3)	28.62	<0.001
Some CH children have to use medication all their lives—answered Yes (%)	130 (50.4)	368 (35.4)	19.68	<0.001
Eating appropriate iodine products during pregnancy can prevent CH to a certain extent—answered Yes (%)	177 (68.6)	547 (52.6)	21.48	<0.001
Genetic counselling can help to prevent CH—answered No (%)	180 (69.8)	419 (40.3)	72.29	<0.001
Score	6.06 ± 2.26	3.11 ± 2.05	20.26	<0.001

FP, false-positive.

Subsequently, the source of knowledge was investigated. Most parents reported that they acquired the relevant knowledge from the Internet, including WeChat public account, Sina Weibo, or Bulletin Board System (*n* = 574, 44.22%), followed by hospitals or doctors (*n* = 318, 24.50%), family members or friends (*n* = 268, 20.65%), publications (*n* = 107, 8.24%), and other sources (*n* = 31, 2.39%). Among parents with enough relevant knowledge of CH, most of them acquired the relevant knowledge from hospitals or doctors than other sources (*n* = 112, 35.22%) (Table 3). Furthermore, the source of knowledge had no statistical difference between the FP group and the control group (*χ^2^ *= 8.143, *P* = 0.086).

**Table 3:** Influence factors of parents’ relevant knowledge of congenital hypothyroidism (CH) [N (%)].

Variable	Knowledge of CH	*χ^2^*	*P*
Age of parents (years)			
<25 (*n* = 352)	84 (23.9)	5.45	0.066
25–35 (*n* = 601)	167 (27.8)		
>35 (*n* = 345)	73 (21.2)		
Sex of parents			
Male (*n* = 677)	168 (24.8)	0.02	0.898
Female (*n* = 621)	156 (25.1)		
Sex of neonates			
Male (*n* = 665)	163 (24.5)	0.09	0.766
Female (*n* = 633)	161 (25.4)		
First child			
Yes (*n* = 993)	234 (23.6)	4.40	0.036
No (*n* = 305)	90 (29.5)		
Ethnicity			
Han (*n* = 652)	163 (25.0)	0.19	0.909
Zhuang (*n* = 595)	147 (24.7)		
Other (*n* = 51)	14 (27.5)		
Years of education			
≤9 (*n* = 474)	97 (20.5)	9.40	0.009
10–14 (*n* = 429)	111 (25.9)		
>14 (*n* = 395)	116 (29.4)		
Living area			
Rural area (*n* = 662)	151 (22.8)	3.34	0.068
Urban area (*n* = 636)	173 (27.2)		
Annual household income (yuan/year)			
<50,000 (*n* = 390)	97 (24.9)	2.04	0.361
50,000–100,000 (*n* = 705)	184 (26.1)		
>100,000 (*n* = 203)	43 (21.2)		
False-positive experience			
Yes (*n* = 258)	140 (54.3)	147.6	<0.001
No (*n* = 1,040)	184 (17.7)		
Source of knowledge			
Hospitals or doctors (*n* = 318)	112 (35.2)	70.57	<0.001
Internet (*n* = 574)	168 (29.3)		
Family member or friends (*n* = 268)	37 (13.8)		
Publications (*n* = 107)	6 (5.6)		
Other (*n* = 31)	1 (3.2)		

To determine the factors associated with having enough relevant knowledge of CH in all parents, we compared the difference between different demographic characteristics, FP experience, and source of knowledge. The FP experience (*χ^2^* = 147.6, *P* < 0.001), source of knowledge (*χ^2^* = 70.57, *P* < 0.001), education year (*χ^2^* = 9.40, *P* = 0.009), and first child or not (*χ^2^* = 4.40, *P* = 0.036) were significantly correlated with having enough relevant knowledge. We took “having enough relevant knowledge” as the independent variable and “FP experience”, “source of knowledge”, “education year”, and “first child” as the dependent variables into logistic regression. As a result, the FP experience and the source of knowledge were the primary influence factors associated with having enough relevant knowledge of CH (both *P*’s < 0.001) (Table 4).

**Table 4:** Logistic regression analysis of the influence factors to parental awareness of relevant knowledge of congenital hypothyroidism (CH).

Items	*B*	SE	Wald	*df*	Sig.	Exp(B)	95% CI for EXP(B)	
							Lower	Upper
First child (yes)	0.428	0.283	2.28	1	0.131	1.534	0.881	2.67
Education year (≤9)			4.79	2	0.247			
Education year (10–14)	0.414	0.301	1.89	1	0.169	1.513	0.838	2.729
Education year (>14)	0.602	0.465	1.68	1	0.195	1.826	0.734	4.541
False-positive experience (yes)	−5.562	0.524	112.84	1	<0.001	0.004	0.001	0.011
Source of knowledge (hospitals or doctors)			39.99	4	<0.001			
Source of knowledge (internet)	−1.41	0.871	2.62	1	0.105	0.244	0.044	1.345
Source of knowledge (family member or friends)	−3.207	0.507	29.97	1	<0.001	0.04	0.015	0.109
Source of knowledge (publications)	−3.475	0.560	31.01	1	<0.001	0.031	0.01	0.093
Source of knowledge (other)	−5.302	0.470	13.21	1	<0.001	0.005	0.002	0.013
Constant	1.478	0.691	4.57	1	0.033	4.383		

### 3.3. Parental response and parental stress

The parents in the FP group received a recall phone call directly from GX-NBSC when the result of the initial newborn screening test was positive. The parents reported that they felt anxious (*n* = 122, 47.29%), followed by panic (*n* = 49, 18.99%), worried (*n* = 49, 18.99%), concerned (*n* = 30, 11.63%), and distrustful (*n* = 13, 5.04%) right after receiving the phone call. Even after receiving information from well-trained pediatricians on the phone call, most parents reported that they only remembered being asked to bring their neonates to the hospital but did not understand why and even could not recall the name of the disease (*n* = 123, 47.67%). A total of 34.50% of parents reported that they understood the situation and what to do during the phone call; some of them searched the internet with the keyword “congenital hypothyroidism” (*n* = 89). The rest of the parents reported that they did not care what the pediatricians said or blindly believed that their neonates had no health issues (*n* = 46, 17.83%). All parents took their neonates for follow-up tests on an average of 3.81 ± 2.59 days after the parents received the phone call.

Because the PSI scores for subjects whose defensive responding index was >10 were included in the analysis, one mother and two fathers were excluded from the FP group and two mothers and three fathers were excluded from the control group. As shown in Table 5, both the mothers and fathers in the FP group reported higher overall stress on the PSI than those in the control group (mothers: *t* = 15.85, *P* < 0.001; fathers: *t* = 11.43, *P* < 0.001). In the FP group, 11 mothers (9.17%) and one father (0.74%) had scores higher than 85, indicating they needed psychological services. However, no parents in the control group had scores above 85. The differences between groups were more pronounced in the total score, parent–child dysfunctional interaction subscales, and difficult child subscales than in the parental distress subscales (*t* = 13.64, 4.260, 11.52, and 10.02, respectively, all *P*’s < 0.001). Most mothers and fathers in the same group showed similar scores on PSI scores; however, the mothers in the FP group had higher scores on the parent–child dysfunction interaction subscale than the fathers (*t* = 2.51, *P* = 0.013). The parents in the FP group who understood the situation well after the recall phone call had lower PSI scores than the others in the FP group (70.7 ± 8.5 vs. 73.9 ± 7.3, *t* = 3.24, *P* = 0.001). The PSI scores did not significantly differ among parents with a different awareness rate of knowledge and demographic characteristics (all *P*’s > 0.05).

**Table 5:** Parenting stress index (PSI) scores for false-positive (FP) and control groups [means ± SD].

Variable	Control group (F/M = 537/498)	FP group (F/M = 135/120)	3 months later (F/M = 101/101)	6 months later (F/M = 86/92)	12 months later (F/M = 65/82)
Total score					
Mothers	61.9 ± 7.2	73.7 ± 7.8***	69.1 ± 8.1***	66.6 ± 7.3***	63.4 ± 7.7
Fathers	63.3 ± 7.6	71.8 ± 8.2***	69.1 ± 7.6***	66.6 ± 8.5***	62.8 ± 6.2
Parental distress subscale					
Mothers	25.1 ± 5.3	28.1 ± 5.0***	26.3 ± 4.9*	25.7 ± 3.8	25.1 ± 5.1
Fathers	25.9 ± 4.9	27.3 ± 5.5**	27.1 ± 5.0*	25.3 ± 4.9	24.3 ± 3.8*
Difficult child subscale					
Mothers	20.7 ± 4.0	25.8 ± 4.8***	23.5 ± 3.9***	22.2 ± 3.0***	20.8 ± 3.3
Fathers	21.3 ± 4.1	25.9 ± 5.2***	24.1 ± 3.4***	23.9 ± 4.0***	21.9 ± 2.5
Parent–child dysfunction interaction subscale					
Mothers	16.1 ± 3.2	19.8 ± 3.7***	19.2 ± 3.4***	18.7 ± 4.1***	17.5 ± 3.1***
Fathers	16.2 ± 2.4	18.6 ± 3.9***	17.9 ± 3.1***	17.4 ± 3.8***	16.6 ± 3.0

Three mothers and five fathers were excluded because their PSI scores were < 10. F, father; M, mother; FP, false-positive.

* *P* < 0.05.

** *P* < 0.01.

*** *P* < 0.001, compared to the control group.

In the follow-up visit, the total PSI and three subscales scores decreased. ANOVA analysis showed that the total score of PSI and parental distress subscales had no statistical difference between fathers and mothers (all *P*’s > 0.05), while difficult child subscales and parent–child dysfunctional interaction subscales had significant differences (*F* = 8.857, *P* = 0.003; *F* = 20.18, *P* < 0.001, respectively); all of them had a significant decrease as time spent (*F* = 52.47, 14.23, 42.04, and 11.08, respectively, all *P*’s < 0.001); none of them had an interaction between gender and time (all *P*’s > 0.05). Compared with the control group, the total PSI scores remained significantly higher at 3 and 6 months afterward (*t* = 7.54, and 4.76, respectively, both *P*’s < 0.001), while there was no statistical difference at 12 months (*P* > 0.05).

## 4. Discussion

To the best of our knowledge, this is the first psychological survey of parents of neonates who had FP NBS results for CH in China. Compared with the parents in the control group, the parents in the FP group had better knowledge of CH and had higher PSI scores. The parenting stress decreased gradually in the FP group; it took possibly 1 year to return to normal. The FP experience and source of knowledge were associated with having the relevant knowledge of CH.

The previous study showed that the relevant knowledge might decrease stress when FP happens (12). In China, public hospitals are also obliged to provide health education on knowledge of NBS to parents. The first time is during the pregnant women's class held by the obstetrics department; the examinations to be done during and after pregnancy will be introduced in the school. The second time is before NBS; clinicians ask parents to sign the informed consent for NBS and interpret the information, including the meaning of NBS, the program of NBS, the process of NBS, the possibility of FP, and retesting. During the recall phone call from GX-NBSC is at least the third time. However, for various reasons, only 24.96% of all participating parents had enough relevant knowledge of CH, which might explain why parents were so stressed about the disease. The possible reason for the lack of knowledge may be due to the low incidence rate of CH, and parents did not pay as much attention to CH as thalassemia or G6PD deficiency. Therefore, sufficient health education, counseling, or support on CH or NBS should be provided to parents of neonates. According to our survey, the FP experience is one of the critical factors. The stress from the FP experience motivated parents to acquire more knowledge of CH, which is very common in education (13). Another factor associated with having relevant knowledge of CH in our survey was the source of knowledge. Although more parents acquired the relevant knowledge from the internet, the parents who received them from hospitals or doctors had higher knowledge scores. The possible reasons are as follows: first, parents may search for the information on the internet right after the recall phone call; second, new media (such as WeChat, Sina Weibo, and Tik-Tok) is easier to be accepted by modern parents. However, the knowledge from the internet may contain misinformation, which is less authentic than the knowledge from hospitals or doctors (14). Therefore, hospitals should play a more important role in targeted health education by using new media methods to increase the knowledge of NBS among all prospective parents. In addition, a quiz could be used after health education to test the learning achievements.

An FP NBS result can passively improve parental knowledge of CH to some extent but lead to other adverse outcomes, such as anxiety and stress in parents of neonates, even after the neonate is confirmed to be in good health by follow-up tests (15). Therefore, another good time for health education is during the recall phone call. Previous studies have shown that improved communication can reduce parental stress and anxiety caused by FP NBS results (16). In our study, common statements of parents in the first recall phone call were “Oh my gosh, what is going on? How did this happen to me?”, “It must be my fault, I pass this disease to my baby.“, ”I'm dying to know what should I do“, ”My child looks fine, you're a liar!”, and “Is that serious?”; we categorized these feelings as panic, anxiety, worry, distrust, and concern, respectively. However, “How this happen to me?” can be distrustful depending on how a parent says it, e.g., with a sneer. In addition, the parents asked many questions about how or whether COVID-19 may affect their babies, which may increase negative emotions. Due to some historical reasons in recent decades [e.g., the “one-child” policy (1982–2015) and high medical costs], the parents were reluctant to be told that their babies were sick or unwell before diagnosis. It gives the pediatricians a dilemma that if they told the parents too much about CH during the phone call, they would be terrified or refuse to talk anymore; if they told them less, they might pay less attention. In current practice, pediatricians only provide ambiguous education to parents before diagnostic confirmation and all verbal. Once the neonates were diagnosed with CH, parents would receive written notes, and pediatricians would explain every detail on the notes. Furthermore, due to the rampant telecom fraud in China, some parents thought the first recall phone from GX-NBSC may be a scam call and did not believe whatever the pediatricians said in our survey (17). In fact, even though the parents did not believe the first phone call in the first place, some of them would call back and ask again, some of them would call the obstetrics department for verification, and some of them would come to the hospital anyway (they might doubt the phone call but they trust the GX-NBSC), and the pediatricians would call the rest of them 2 days later to make sure they could get medical service in time. Therefore, pediatricians must improve their communication skills, enhance their knowledge, and react fast to make the parents believe them on the phone.

Previous studies have demonstrated that the PSI scores of mothers of neonates with FP NBS results for cystic fibrosis were higher than those of the control group, and some psychosocial responses could not be detected until 1 year later (18). Similarly, we found that the parenting stress faded gradually to normal in 12 months. In the parents of 173 infants with FP results and the parents of 67 children with normal screening results, Gurian et al. found that the FP results of expanded NBS may affect parental stress and parent–child relationships (16). Studies indicate that high-quality parental education and communication may have a positive impact on parents’ stress and anxiety, including those who receive FP results (19). Similar to a previous study, we found that the parents in the FP group who were well-informed during the recall phone call had lower PSI scores. The difference in voice, speed, and tone of speech may reflect clinical manifestations in parents with different scores of PSI. Parents in the FP group had more questions about their neonates, while the parents in the control group had a more relaxed conversation in the interview. In a previous study in China, the parents of neonates with FP results of expanded NBS had higher PSI scores than those in the normal screening result group, but the demographic differences in the PSI scores have never been studied (9). Unfortunately, we did not find any effects of the demographic characteristics of parents in Guangxi on the PSI scores. In our study, we found that the PSI scores were higher in the parents of the FP group, which is consistent with the results of previous studies (15, 18, 19). Moreover, the FP results affected parent–child relationships to a greater extent among mothers than fathers, possibly because of the gender difference.

Our study has some limitations. First, some parents met the inclusion criteria but refused to participate in our investigation, which may have induced selection bias. Second, the PSI score of parents whose neonates had true positive results were missing. In our presurvey, those parents were rare and unwilling to cooperate; therefore, they were not included in our study. Third, due to the COVID-19 pandemic, we could not investigate the parents directly, and about half of them were lost to follow-up. In addition, the questions about knowledge of CH were more like a quiz than a questionnaire, and the threshold of correctness was decided before the survey. Therefore, we need to make our questionnaire and cutoff value more reasonable in further studies.

## 5. Conclusions

The results of this study suggest that parents in Guangxi had higher PSI scores when their neonates had FP CH results, and it may take 12 months to get back to normal levels. The FP experience improved the relevant knowledge of CH among parents, but it also had some negative consequences. Overall, with the “three-child” policy (2021–) and the progress of medical insurance in recent years, the parents may have more chance to understand these disease, and targeted health education should be provided in good timing with a good strategy.

## Data Availability

The original contributions presented in the study are included in the article, further inquiries can be directed to the corresponding authors.
